# Attitudes, Beliefs, Barriers, and Facilitators of Emergency Department Nurses Toward Patients with Opioid Use Disorder and Naloxone Distribution

**DOI:** 10.5811/westjem.18020

**Published:** 2024-05-21

**Authors:** Collin Michels, Thomas Schneider, Kaitlin Tetreault, Jenna Meier Payne, Kayla Zubke, Elizabeth Salisbury-Afshar

**Affiliations:** * University of Wisconsin, School of Medicine and Public Health, BerbeeWalsh Department of Emergency Medicine, Madison, Wisconsin; † University of Wisconsin, School of Medicine and Public Health, Madison, Wisconsin; ‡ University of Wisconsin, School of Medicine and Public Health, Department of Biostatistics and Medical Informatics, Madison, Wisconsin; § University of Wisconsin, School of Medicine and Public Health, Department of Family Medicine and Community Health, Madison, Wisconsin; ∥ University of Wisconsin, School of Medicine and Public Health, Department of Population Health Sciences, Madison, Wisconsin

## Abstract

**Introduction:**

As opioid overdose deaths continue to rise, the emergency department (ED) remains an important point of contact for many at risk for overdose. In this study our purpose was to better understand the attitudes, beliefs, and knowledge of ED nurses in caring for patients with opioid use disorder (OUD). We hypothesized a difference in training received and attitudes toward caring for patients with OUD between nurses with <5 years and ≥6 years of clinical experience.

**Methods:**

We conducted a survey among ED nurses in a large academic medical center from May–July 2022. All ED staff nurses were surveyed. Data entry instruments for the nursing surveys were programmed in Qualtrics, and we analyzed results R using a chi-square test or Fisher exact test to compare nurses with <5 years and ≥6 years of clinical experience. A *P*-value of < 0.05 was considered statistically significant.

**Results:**

We distributed 74 surveys, and 69 were completed (93%). Attitudes toward naloxone distribution from the ED were positive, with 72% of respondents reporting they were “very” or “extremely” supportive of distributing naloxone kits to individuals at risk of overdose. While attitudes were positive, barriers included limited time, lack of system support, and cost. Level of comfort in caring for patients with OUD was high, with 78% of respondents “very” or “extremely” comfortable. More education is needed on overdose education and naloxone distribution (OEND) with respondents 38% and 45% “a little” or “somewhat” comfortable, respectively. Nurses with <5 years of experience reported receiving more training on OEND in nursing school compared to those with ≥6 years of experience (*P* = 0.03). There were no significant differences in reported attitudes, knowledge, or comfort in caring for patients with OUD.

**Conclusion:**

In this single-center survey, we found ED nurses were supportive of overdose education and naloxone distribution. There are opportunities for targeted education and addressing systemic barriers to OEND. All interventions should be evaluated to gauge impact on knowledge, attitudes, and behaviors.

Population Health Research Capsule What do we already know about this issue?

*Emergency departments play a crucial role in caring for patients with opioid use disorder (OUD) with interventions such as overdose education and naloxone distribution.*

What was the major research question?

*What are attitudes of ED nurses related to caring for patients with OUD, and training in overdose education and naloxone distribution (OEND)?*

What is the major finding of the study?

*ED nurses have positive attitudes (72%) toward naloxone distribution. Early career nurses (<5 years) had more OEND training.*

How does this study improve population health?

*Results highlight opportunities for targeted nursing education, addressing barriers and facilitators to OEND in the ED, thereby improving care for patients with OUD.*

## INTRODUCTION

Opioid use disorder (OUD) is associated with a 20-fold risk of early death due to overdose, infection, trauma, or suicide.^1^ Nationally, an estimated 68,000 people died of opioid-related overdose in 2020, and 2.7 million suffered from OUD.^2^ The impact of non-medical opioid use and OUD can be seen in many healthcare settings, including the emergency department (ED), as opioid-related visits in the ED had an estimated cost of $1.47 billion per year between 2016–2017.^2^^,^^3^

Patients presenting to the ED for opioid-related encounters, including opioid overdose, are at high risk for negative outcomes. Emergency department-based interventions such as overdose education and naloxone distribution (OEND) can have a significant impact on opioid-related morbidity and mortality. Naloxone is an opioid receptor antagonist that is used to quickly reverse the effects of opioid overdose. In 2018, the US Surgeon General recommended increasing access to naloxone for those who are at an increased risk of an opioid overdose.^4^ The American College of Emergency Physicians also recommends providing naloxone for patients at increased risk of opioid overdose, including those discharged from the ED after an opioid-related visit as well as any patient with a history of OUD.^5^

Emergency department-based take-home naloxone programs have been an effective means of distributing naloxone to patients at risk for future overdose^6^^,^^7^; and OEND from the ED has been shown to have positive impact on trained laypersons in addition to patients and their social network.^8^ Large-scale OEND has been shown to be an effective public health intervention.^9^ Patient education related to overdose prevention and naloxone distribution can be provided by ED nurses who routinely spend more time with patients than the treating clinician. Clinical nurse specialist-led OEND in the ED have been effective across an integrated healthcare system.^10^ While much is known about the beliefs, attitudes, and barriers of prescribers toward naloxone distribution, including time, cost, and clinical decision support, less is known about nurse perspectives in the ED.^6^^,^^7^^,^^11^^–^^15^ We sought to evaluate nurse attitudes, beliefs, barriers, and facilitators to naloxone distribution in an academic ED in the Midwest.

## METHODS

From May–July 2022 we conducted a survey of ED nurses at a quaternary-care, academic ED in the Midwest that sees approximately 60,000 patients per year. The research team, which included an emergency physician and an addiction medicine physician, created a survey tool in collaboration with survey methodology experts from the University of Wisconsin Survey Center. Most items on the survey were developed by the team, but the stigma questions were adapted from a validated mental health stigma survey.^15^^–^^17^ Research coordinators in the ED distributed 74 paper surveys to full and part-time ED staff nurses at daily staff huddles during the study period. Each respondent was allowed to complete only one survey. A $5 pre-incentive was included with the survey at the time of distribution.

We used a chi-square test or Fisher exact test to assess the difference in nurse attitudes, based on relative job experience (≤5 years v ≥6 years), regarding perception, knowledge, and barriers for naloxone distribution and caring for patients with OUD. All analyses were done in R v 4.1.1 2021 (R Foundation for Statistical Computing, Vienna, Austria). A *P*-value of <0.05 was considered statistically significant.

### Disclosures

This study was reviewed by the University of Wisconsin-Madison Minimal Risk Research Institutional Review Board and deemed exempt. None of the authors have any financial conflicts of interest to disclose.

## RESULTS

Surveys were distributed to 74 ED nurses, with a 93% response rate. Respondents had a breadth of clinical experience, with 60% having been a practicing nurse for six years or more. Of that group, 21% had been a practicing nurse for ≥16 years. The majority of the ED nurses reported completing their nursing training in the Midwest (83%). Other regions represented were the West (7.6%), Southwest (1.5%), Southeast (4.5), and Northeast (3%).

Overall, the level of training on OEND during nursing school was low, with 77% reporting no or a little education received. Nurses with 0–5 years of experience reported receiving more education compared to nurses with ≥6 years of experience (*P* = 0.03). When asked about level of comfort providing education related to naloxone for overdose prevention immediately following nursing school, 67% felt “not at all” or “only a little” prepared. Despite more recent nursing school graduates reporting more education in nursing school, there were no differences in how prepared they felt to provide OEND (*P* = 0.63).

Responses were mixed when they were asked about the perceived effectiveness of naloxone kits as a public health intervention, with 55% of all nurses reporting naloxone kits are “a little” or “somewhat” effective. However, the majority (66%) felt that naloxone kits would not increase behavior that put people at risk for overdose. Additional responses to questions about attitudes, beliefs, barriers, and facilitators to naloxone distribution from the ED are available in the Table. Responses to all questions were compared between the nurses with 0–5 years’ experience to those with ≥6 years’ experience, and no statistically significant differences were appreciated.

**Table.** Responses of emergency department nurses to questions about attitudes, beliefs, barriers, and facilitators to naloxone distribution from the ED.

		Not at all	A little	Somewhat	Very	Extremely
Attitudes	How much do you support giving naloxone kits to individuals who might be at risk for opioid overdose?	1.5% (1)	10.3% (7)	16.2% (11)	29.4% (20)	42.6% (29)
	How effective is giving a naloxone kit to people who use drugs as a public health intervention?	0.0% (0)	24.6% (17)	30.4% (21)	29.0% (20)	14.5% (10)
	How likely is giving a naloxone kit to people who use drugs going to lead to behaviors that increase risk for overdose, eg, using more opioids or using in combination with other drugs?	41.8% (28)	23.9% (16)	25.4% (17)	9.0% (6)	0.0% (0)
Comfort	Asking screening questions about non-prescribed opioid use?	0.0% (0)	2.9% (2)	10.1% (7)	47.8% (33)	39.1% (27)
	Caring for patients who use non-prescribed opioids?	0.0% (0)	1.4% (1)	20.3% (14)	46.4% (32)	31.9% (22)
	Offering a naloxone kit to be able to reverse an overdose?	1.4% (1)	5.8% (4)	33.3% (23)	31.9% (22)	27.5% (19)
	Teaching a layperson to administer naloxone?	2.9% (2)	10.1% (7)	27.5% (19)	34.8% (24)	24.6% (17)
	Providing care to a person with an opioid use disorder compared to helping a person with a physical illness?	3.0% (2)	4.5% (3)	26.9% (18)	47.8% (32)	17.9% (12)
	Educating patients about opioid overdose prevention?	0.0% (0)	5.8% (4)	36.2% (25)	44.9% (31)	13.0% (9)
	Educating patients about overdose response and naloxone administration?	4.3% (3)	15.9% (11)	29.0% (20)	37.7% (26)	13.0% (9)
	Educating patients about overdose prevention?	2.9% (2)	18.8% (13)	30.4% (21)	34.8% (24)	13.0% (9)
Barriers	Limited staff time?	0.0% (0)	4.5% (3)	18.2% (12)	30.3% (20)	47.0% (31)
	Lack of systems supporting it to happen in a time efficient way?	1.5% (1)	4.6% (3)	21.5% (14)	43.1% (28)	29.2% (19)
	Lack of clinical decision support to ensure consistent process?	31.% (2)	10.9% (7)	25.0% (16)	39.1% (25)	21.9% (14)
	How much of a barrier to dispensing naloxone kits from the ED is lack of insurance or limited insurance coverage leading to high costs to patients?	9.4% (6)	10.9% (7)	17.2% (11)	39.1% (25)	23.4% (15)
	Concerns about being able to identify patients at risk for overdose?	21.2% (14)	28.8% (19)	40.9% (27)	9.1% (6)	0.0% (0)
	Concerns that a layperson won’t be able to administer it appropriately?	28.8% (19)	37.9% (25)	27.3% (18)	6.1% (4)	0.0% (0)
	Concerns that providing a naloxone kit will lead to more or riskier drug use?	48.5% (32)	15.2% (10)	16.7% (11)	18.2% (12)	1.5% (1)
	Concerns that patients will be offended by it being offered?	40.9% (27)	19.7% (13)	31.8% (21)	6.1% (4)	1.5% (1)
Facilitators	Funding to ensure patients don’t have to pay co-pays for cost of the naloxone kit?	3.1% (2)	7.8% (5)	20.3% (13)	32.8% (21)	35.9% (23)
	Clinical decision support that makes the prescription an automated process?	0.0% (0)	9.4% (6)	26.6% (17)	48.4% (31)	15.6% (10)
	Education for staff?	1.6% (1)	3.1% (2)	43.8% (28)	35.9% (23)	15.6% (10)
	How much of a facilitator to discharging a patient from the ED with a naloxone kit is patient education materials to teach about overdose prevention and naloxone administration?	3.2% (2)	7.9% (5)	27.0% (17)	44.4% (28)	17.5% (11)

*ED*, emergency department.

Overall comfort level for caring for patients who use non-prescribed opioids was high, with 78% of respondents very or extremely comfortable. Again, no differences were appreciated between nurses with 0–5 years’ experience and those with ≥6 years’ experience.

Barriers and facilitators to naloxone distribution in the ED are varied and related to time, education, and cost concerns. Staff reported the most significant barrier was limited staff time, with 47% reporting this was an “extremely” impactful barrier. These are similar to previously described barriers and facilitators that prescribers report facing; responses are included in the Table.^14^^–^^18^

## DISCUSSION

Emergency department nurses are critical to the effectiveness of ED-based OEND programs. Although there have been multiple studies looking at emergency clinician attitudes, beliefs, barriers, and facilitators to naloxone distribution, little is known about ED nurse-specific factors for OEND. Although nurses in practice for ≤5 years reported receiving more education on naloxone for overdose prevention while in nursing school, the additional education did not relate to statistically significant differences in attitudes, comfort, or perceived barriers or facilitators. Further research is needed to provide a better understanding of why receiving more education did not lead to increased comfort or knowledge and whether offering more targeted education can improve these metrics. Despite receiving more education, early career nurses have had less experience caring for patients with OUD, which may have contributed to the results.

Overall, most respondents were comfortable caring for patients with OUD, including asking OUD screening questions. Slightly less than half felt naloxone is a “very” or “extremely” effective public health intervention, which is an important area for future educational efforts and evaluation. Additional areas for educational foci include trainings on overdose prevention education and naloxone training for patients and their friends/family while in the ED. This data provides a baseline understanding and can be re-assessed after further educational initiatives.

We found nursing-identified barriers were similar to previously described prescriber barriers including limited time, cost, and lack of efficient system support.^18^^–^^20^ Some of these barriers can be addressed with clinical decision support, including prompts to order naloxone for patients with opioid-related diagnostic codes. Providing standardized, easy-to-follow instructions on overdose prevention and naloxone administration can benefit both the patients and the staff member providing the education. Although handouts are helpful, regular education by content experts would provide continued education to ensure all staff are comfortable with overdose prevention education and naloxone use moving forward.

Overall, ED nurses were open to receiving more education, and most nurses identified this as a facilitator to expanding naloxone distribution in the ED. Using baseline surveys like the one our team used can guide ED leadership when developing educational and systems interventions for nursing staff.

## LIMITATIONS

Limitations of this study include evaluating a single, academic Level I trauma center; so results may not apply more broadly to other EDs. We did not evaluate for nursing experience in areas outside the ED. Additionally, the number of ED nurses surveyed was small (69); so it is possible that the sample size was too small to enable us to identify differences between the nurses with less experience as compared to those with more experience.

## CONCLUSION

Understanding attitudes, beliefs, barriers, and facilitators of naloxone distribution among ED nurses is important for successful implementation of overdose education and prevention programming. Emergency department nurses surveyed were generally supportive of naloxone distribution and comfortable caring for patients with OUD. There are opportunities for addressing systemic barriers and providing targeted education to facilitate ED-based naloxone distribution. These results show opportunities to improve care for patients with OUD, although future research is needed to determine whether education impacts knowledge, attitudes, and behaviors.

## References

[1] National Academies of Sciences, Engineering, and Medicine . Medications for Opioid Use Disorder Save Lives. Washington DC: The National Academies Press, 2019. 30896911

[2] Substance Abuse and Mental Health Services Administration . Key Substance Use and Mental Health Indicators in the United States: Results from the 2020 National Survey on Drug Use and Health. 2022. Available at: https://www.samhsa.gov/data/sites/default/files/reports/rpt35325/NSDUHFFRPDFWHTMLFiles2020/2020NSDUHFFR1PDFW102121.pdf. Accessed January 20, 2023.

[3] Weiner SG Baker O Bernson D et al . One-year mortality of patients after emergency department treatment for nonfatal opioid overdose. Ann Emerg Med. 2020;75(1):13–7. 31229387 10.1016/j.annemergmed.2019.04.020 PMC6920606

[4] Office of the Surgeon General . U.S Surgeon General’s Advisory on Naloxone and Opioid Overdose. 2022. Available at: https://www.hhs.gov/surgeongeneral/reports-and-publications/addiction-and-substance-misuse/advisory-on-naloxone/index.html. Accessed January 10, 2023.

[5] Duber HC Barata IA Cioè-Peña E et al . Identification, management, and transition of care for patients with opioid use disorder in the emergency department. Ann Emerg Med. 2018;72(4):420–31. 29880438 10.1016/j.annemergmed.2018.04.007 PMC6613583

[6] Eswaran V Allen KC Cruz DC et al . Development of a take-home naloxone program at an urban academic emergency department. J AM Pharm Assoc. 2020;60(6):324–31. 10.1016/j.japh.2020.06.017 32690447

[7] Eswaran V Allen KC Bottari DC et al . Take-home naloxone program implementation: lessons learned from seven chicago-area hospitals. Ann Emerg Med. 2020;76(3):318–27. 32241746 10.1016/j.annemergmed.2020.02.013

[8] Dwyer K Walley A Langlois B et al . Opioid education and nasal naloxone rescue kits in the emergency department. West J Emerg Med. 2015;16(3):381–4. 25987910 10.5811/westjem.2015.2.24909 PMC4427207

[9] Walley AY Xuan Z Hackman HH et al . Opioid overdose rates and implementation of overdose education and nasal naloxone distribution in Massachusetts: interrupted time series analysis. BMJ. 2013;346:f174. 23372174 10.1136/bmj.f174 PMC4688551

[10] Mullennix SC Iseler J Kwiatkowski GM et al . A clinical nurse specialist-led emergency department naloxone distribution program. Clin Nurse Spec. 2020;34(3):116–23. 32250993 10.1097/NUR.0000000000000515

[11] Lowenstein M Kilaru A Perrone J et al . Barriers and facilitators for emergency department initiation of buprenorphine: a physician survey. Am J Emerg Med. 2019;37(9):1787–90. 30803850 10.1016/j.ajem.2019.02.025 PMC7556325

[12] Hawk KF D’Onofrio G Chawarski MC et al . Barriers and facilitators to clinician readiness to provide emergency department-initiated buprenorphine. JAMA Netw Open. 2020;3(5):e204561. 32391893 10.1001/jamanetworkopen.2020.4561 PMC7215257

[13] Ellis K Walters S Friedman SR et al . Breaching trust: a qualitative study of healthcare experiences of people who use drugs in a rural setting. Front Sociol. 2020;5:593925. 33869521 10.3389/fsoc.2020.593925 PMC8022503

[14] Lacroix L Thurgur L Orkin AM et al . Emergency physicians’ attitudes and perceived barriers to the implementation of take-home naloxone programs in Canadian emergency departments. CJEM. 2018;20(1):46–52. 28918769 10.1017/cem.2017.390

[15] Kassam A Papish A Modgill G et al . The development and psychometric properties of a new scale to measure mental illness related stigma by health care providers: the Opening Minds Scale for Health Care Providers (OMS-HC). BMC Psychiatry. 2012;12:62. 22694771 10.1186/1471-244X-12-62 PMC3681304

[16] Ezell JM Walters S Friedman SR et al . Stigmatize the use, not the user? Attitudes on opioid use, drug injection, treatment, and overdose prevention in rural communities. Soc Sci Med. 2021;268:113470. 33253992 10.1016/j.socscimed.2020.113470 PMC7755701

[17] Kelly T Hawk K Samuels E et al . Improving uptake of emergency department-initiated buprenorphine: barriers and solutions. West J Emerg Med. 2022;23(4):461–7. 35980414 10.5811/westjem.2022.2.52978 PMC9391022

[18] Barbour K McQuade M Somasundaram S et al . Emergency physician resistance to a take-home naloxone program led by community harm reductionists. Am J Emerg Med. 2018;36(11):2110–2. 29588147 10.1016/j.ajem.2018.03.036

[19] Penm J MacKinnon NJ Lyons MS et al . Combatting opioid overdoses in Ohio: emergency department physicians’ prescribing patterns and perceptions of naloxone. J Gen Intern Med. 2018;33(5):608–9. 29492780 10.1007/s11606-018-4353-6 PMC5910359

[20] Lane BH Lyons MS Stolz U et al . Naloxone provision to emergency department patients recognized as high-risk for opioid use disorder. Am J Emerg Med. 2021;40:173–6. 33243535 10.1016/j.ajem.2020.10.061

